# Alteration of rocks by endolithic organisms is one of the pathways for the beginning of soils on Earth

**DOI:** 10.1038/s41598-018-21682-6

**Published:** 2018-02-20

**Authors:** Nikita Mergelov, Carsten W. Mueller, Isabel Prater, Ilya Shorkunov, Andrey Dolgikh, Elya Zazovskaya, Vasily Shishkov, Victoria Krupskaya, Konstantin Abrosimov, Alexander Cherkinsky, Sergey Goryachkin

**Affiliations:** 1 Institute of Geography, Russian Academy of Sciences, Department of Soil Geography and Evolution, Moscow, 119017 Russia; 2 TU München, Lehrstuhl für Bodenkunde, Freising-Weihenstephan, 85354 Germany; 3 Institute of Geology of Ore Deposits, Petrography, Mineralogy and Geochemistry, Russian Academy of Sciences, Laboratory of Crystal Chemistry of Minerals, Moscow, 119017 Russia; 4 V.V. Dokuchaev Soil Science Institute, Russian Academy of Sciences, Department of Soil Physics, Hydrology and Erosion, Moscow, 119017 Russia; 5 Center for Applied Isotope Studies, University of Georgia, Athens, 30602 United States

## Abstract

Subaerial endolithic systems of the current extreme environments on Earth provide exclusive insight into emergence and development of soils in the Precambrian when due to various stresses on the surfaces of hard rocks the cryptic niches inside them were much more plausible habitats for organisms than epilithic ones. Using an actualistic approach we demonstrate that transformation of silicate rocks by endolithic organisms is one of the possible pathways for the beginning of soils on Earth. This process led to the formation of soil-like bodies on rocks *in situ* and contributed to the raise of complexity in subaerial geosystems. Endolithic systems of East Antarctica lack the noise from vascular plants and are among the best available natural models to explore organo-mineral interactions of a very old “phylogenetic age” (cyanobacteria-to-mineral, fungi-to-mineral, lichen-to-mineral). On the basis of our case study from East Antarctica we demonstrate that relatively simple endolithic systems of microbial and/or cryptogamic origin that exist and replicate on Earth over geological time scales employ the principles of organic matter stabilization strikingly similar to those known for modern full-scale soils of various climates.

## Introduction

The pedosphere emergence is attributed to the most ancient forms of terrestrial life in the Early Precambrian which strongly aided the abiotic decay of rocks. How could the first progenitors of soils look like when life consistently started to colonize silicate hard rocks on land and what are the best natural models we have in hand nowadays to approximate these protosoils?

Although the new knowledge on Proterozoic and Archaean paleosols^1–4^ is continuously arising the understanding of initial stages of soil production from hard rock in a prokaryotic biosphere is scarcely replenished by the new data due to extremely low preservation potential of these early phases. Initial soil-like bodies containing biotic components possibly emerged from cyanobacteria and archaea organism-to-mineral interactions accompanied by an ensemble of abiotic weathering agents. Cyanobacteria as the first biotic component in ecosystems on land are evidenced by microfossil and geochemical records including organic molecular signatures that date back to at least 2.7 Gyr ago (Ga)^5,6^. The fossil land plants and fungi appeared ~0.48–0.46 Ga, whereas protein sequence analyses indicated that green algae and fungi were already present by 1 Ga and land plants appeared by 0.7 Ga^7^. Thus, for nearly 2 Gyr^8^ the terrestrial organic carbon (OC) pools were fueled by cyanobacteria, algae and lichens on later stages, and lacked any contribution from vascular plants arguably known as main sources of soil organic matter. The soils formed through interactions with these cryptogamic and microbial photoautotrophs were probably among the most long-living types of bio-abiotic systems in the Earth’s history^9^. What were the pathways for initial organic matter (OM) build up in these soils, as well as the mechanisms of organo-mineral interactions? The answer to this question helps to understand how OM stabilization principles evolved on Earth and provide the baseline from the past to address the “hot” issue of OC stabilization in much younger paleosols^10^ and modern soils^11,12^ both with more advanced organo-mineral matrix complicated by OM from vascular plants, geological and weathering background and other variables.

The present biocrust varieties^13^ especially dominated by cyanobacteria maintain remarkable similarities to the Precambrian ones. Biofilms and biocrusts on hard rocks continue to be exposed to such ancient stresses as solar radiation, desiccation and re-hydration, temperature fluctuations, oligotrophy and others^14^, yet of different intensity. Many modern endolithic communities (live inside the rocks) can tolerate environmental extremes that make them strong candidates for the colonization of early Earth and planetary surfaces^15–17^. We propose that modern subaerial endolithic systems (comprise endolithic organisms, mineral environment they inhabit and *in situ* altered organo-mineral byproducts^18^) of East Antarctica provide the closest appropriate proxy to render the soils progenitors in Precambrian when firstly cyanobacteria and much later fungi and lichens started to colonize silicate hard rocks. This is most relevant to the cryptoendolithic varieties. Right from the times when subaerial niches on hard rocks experienced high UV-radiation levels (especially before screening pigments were obtained through evolution^19^) and other stresses the porous interior of rocks was a much more secure place to sustain life also enabling better nutrient and more constant water supply. Thus, endolithic biofilms are more plausible settlers than epilithic ones. Just as in regions with harsh environment nowadays, e.g. Antarctica, where extreme conditions create on the rock surface a barrier to colonization that cannot be overcome by physiological adaptations^15^ while the pore space in sandstones, granitoids and gneisses provide milder microenvironments^20^. The ice-free areas of East Antarctica being the most climatically extreme and isolated environments on Earth are unique natural reserves of intact cryptogamic covers, thus represent landscapes of the first choice to explore analogues of ancient subaerial biocrusts and biofilms including endolithic ones. Although East Antarctica is different from more warm places where one could assume the first soils on Earth, it offers the unique setting of bare storm prone geological surfaces and high UV-radiation while at the same time water is scarce.

Some signs of endolithic organisms are recorded in mineral substrates of Proterozoic and Archaean^17,21^, however they are mainly attributed to the euendolithic biota of the aqueous environment which employ microboring strategies. The *in situ* fossil record of the Precambrian *subaerial* endolithic systems (especially cryptoendolithic ones) could be very poor if any due to self-destructive^22^ and product-dispersive^23^ trend in development, location at hard rock exposures but not in sedimentary basins.

Contemporary subaerial endolithic systems from extreme environments that we report in this study have a great potential to provide insights into early principles of organo-mineral interactions and soil production from hard rocks. They lack noise originating from vascular plants with root systems and bryophytes, thus enable to look at biota-to-mineral interactions of organisms with very old phylogenetic ages (cyanobacteria, fungi, lichen) in “pure” and “pristine” conditions. The closest approach was recently applied by Mitchell *et al*.^24^ who investigated soil development under modern cryptogamic ground covers in Iceland but with more advanced organisms involved e.g. bryophytes, thus relevant to understanding of vegetation and soils “coevolution” mainly in later times starting from Paleozoic.

The knowledge obtained from endolithic organisms most often was used in developing scenarios for exobiology or understanding early life, but not soils. Johnston and Vestal^25^ first noted that zonation in rocks colonized by cryptoendolithic lichen resembles soil horizons from temperate humid climates. Later Mergelov *et al*.^18^ developed this analogy with Podzols (Spodosols) and proposed to consider endolithic systems as the soil-like bodies. As the word “soil” in English language is conventionally employed for the loose material, we suggest the term soloid^26^ (from Latin *solum* soil and Greek *eîdos*, likeness) to be applied to the endolithic systems formed *in situ* on hard rocks. Soloids were probably the first protosoils on hard rocks and thus on terrestrial ground.

To unravel the structure and composition of these micro scale modern *subaerial cryptoendolithic systems* on silicate rocks of Antarctica we used a set of analytical techniques including NanoSIMS, with a special emphasize on OM accumulation and stabilization. Previous, yet few, NanoSIMS studies concerned microborings of *euendolithic* organisms and tested biogenicity of microfossils in more geological settings^27–29^.

We are now able (1) to reconcile existing knowledge on organo-mineral interactions and mechanisms of OC stabilization in subaerial endolithic systems within the soil science paradigm; (2) to evaluate endolithic systems as proxies of the first soils developed *in situ* from hard rocks in Precambrian and (3) by applying actualistic approach and the data on endolithic systems from East Antarctica to demonstrate that from the very first appearance of soils the processes at work were possibly very much the same as the present ones.

## Results and Discussion

### Soil-like patterns of endolithic systems

**Figure 1:**
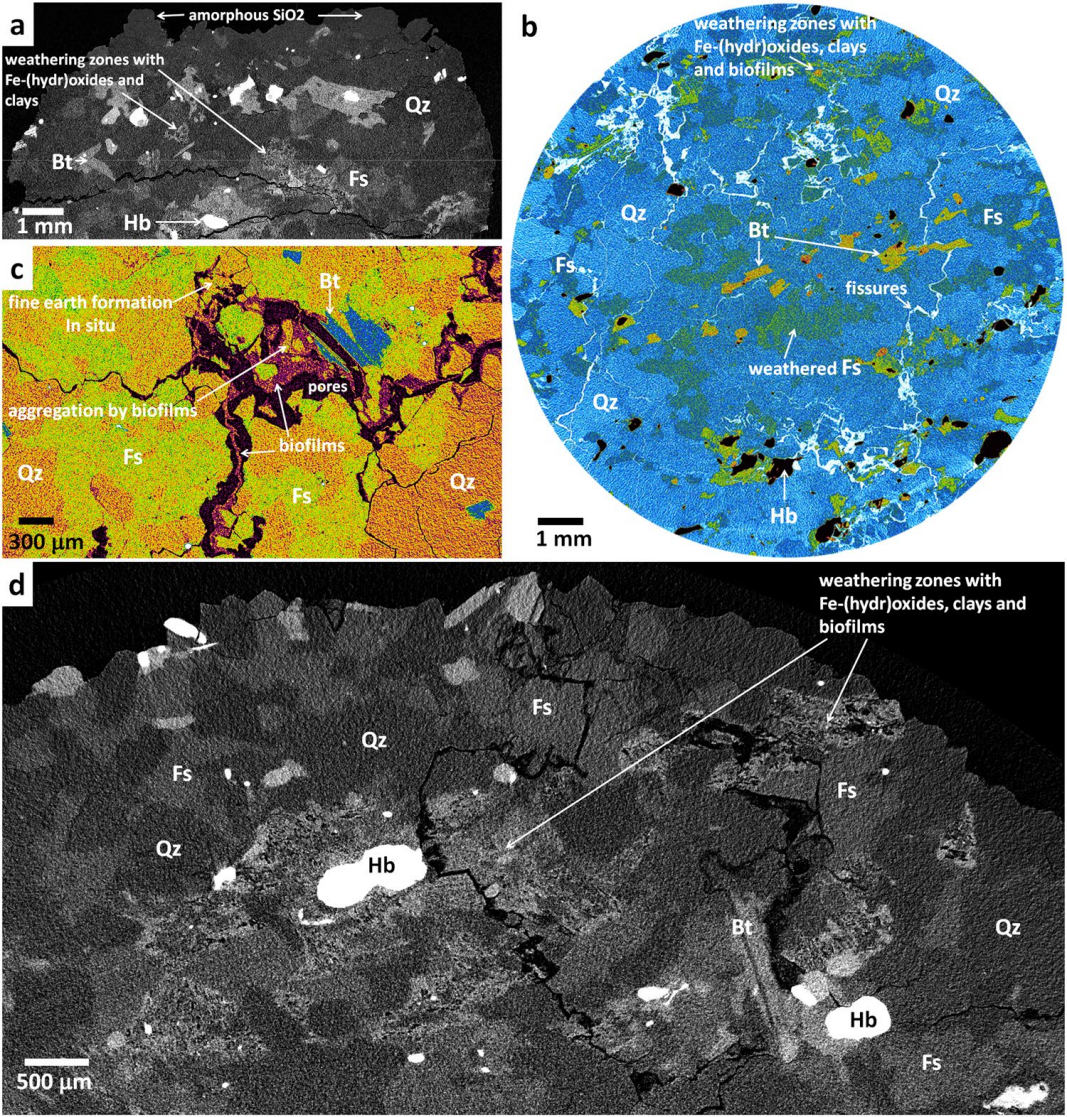
Structure of endolithic system on gneiss as seen by µCT: (**a**) – vertical cross-section, (**b**) – horizontal cross-section, inverted false colors, the fissures and pores appear white, (**c**) – horizontal cross-section, pocket with biofilms and fine earth formed *in situ*, false colors, the fissures and pores appear black, (**d**) – horizontal cross-section, visualization of weathering zones with Fe-(hydr)oxides, clay minerals, both in close vicinities with biofilms. Qz – quartz, Fs – feldspars, Bt – minerals of biotite series, Hb – hornblende. Images b and c were deliberately processed in different color palletes to achieve better visualization of the fine details.

According to µCT data different layers of endolithic systems (we refer here to the cryptoendolithic varieties) on granitoids and gneisses in oases of East Antarctica are intensively connected by fractures. These subtle branched subvertical (<2.5–20 microns) and larger perforating subhorizontal fractures (20–200 microns) serve as a uniform network (Fig. 1a,b) making the rock permeable for dissolved products of endolithic weathering. Thus, these rocks act more like a very dense soil rather than a sealed hard rock. The structure of endolithic system itself is also largely determined by the inhabiting organisms. Loose filaments and cell clusters grow in pore spaces between and around the minerals so that even such organisms as lichens are completely embedded in the rock matrix covered by the hard surface crust. The weathering zones of 0.1–1.0 mm size are formed and can be distinguished by µCT due to the well-established grid of microfractures and numerous roundish pores creating a “perforated” microcellular pattern (Fig. 1d). These zones show clear indications for organo-mineral associations (see also SEM-EDX and NanoSIMS data), pointing to the fact that this OC stabilization mechanism highly important in common soils also operates in endolithic systems. At the same time initial stages of *in situ* fine earth formation were detected in pocket-like microzones (Fig. 1c) enriched in biofilms. Fine earth production - that what really converts rock to soil.

**Figure 2:**
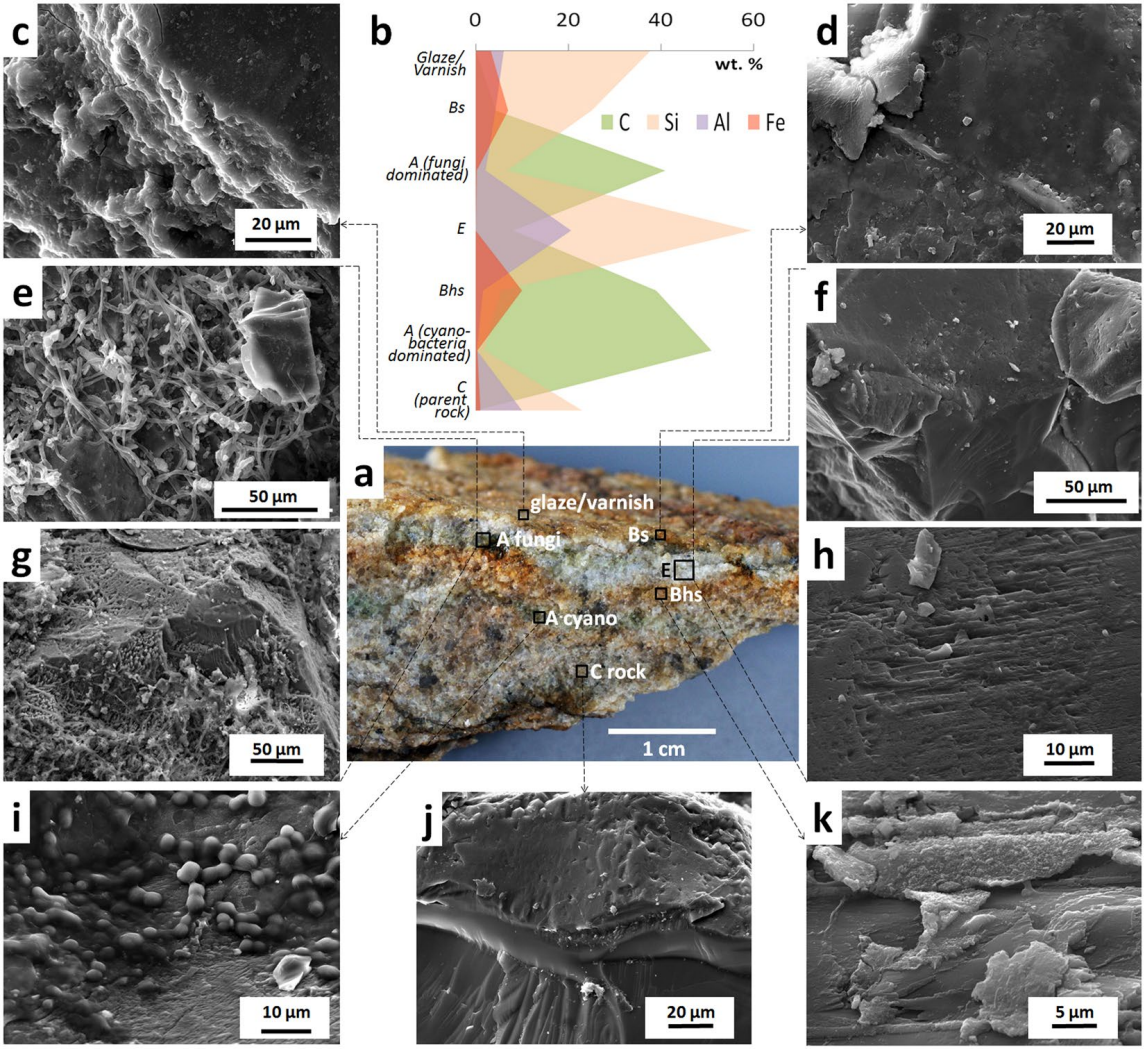
Soil-like eluvial-illuvial differentiation in cryptoendolithic system (Thala Hills, East Antarctica, gneiss): (**a)** – vertical stratification in endolithic system, the sequence of horizons resembles the profile of Podzol, for convenience the indexes of horizons are given by the analogy with the ones in Podzols; (**b**) – vertical distribution of C, Si, Al, Fe according to SEM-EDX elemental analyses in thin section from the endolithic system; (**c**) – silica glaze and varnish on the day surface of the gneiss rock; (**d**) – the upper Bs horizon (sesquioxides accumulation) with iron rich coatings; (**e**,**g**) – endolithic organogenous horizon A dominated by fungi with weathering patterns associated with melanin and hyaline pigmented fungal hyphae; (**f**,**h**)– bleached eluvial horizon E, clean surfaces without organo-mineral coatings and mineral degradation patterns; (**i**) – endolithic organogenous horizon dominated by cyanobacteria biofilms; (**j**) – rather clean mineral surfaces in the parent rock without distinct biofilms; (**k**) – the lower Bhs horizon (organic matter and sesquioxides accumulation) with carbon and iron rich coatings. Images (**c**–**k**) made by SEM in secondary electrons mode. Fungi and cyanobacteria dominated horizons may be linked by the mutualistic relationships of inhabiting organisms, endolithic (proto)lichen formation is not excluded.

Interactions between myco- and photobionts of endolithic lichens possibly occur along fissure networks as observed in µCT. These networks drive the elemental transfers in the interior of the upper 1–2 cm of granitoids and gneisses and in favorable conditions enable an eluvial-illuvial differentiation of the lichen and fungi dominated endolithic systems (for details see Fig. 2). The network of these fine fractures connects zones depleted in Fe at the center of endolithic system with the oxidized Fe-enriched loci and layers at the top and bottom. Mobilization and deposition of Fe occur during rare wetting events and subsequent desiccation of the rock surface in Antarctica; thermogradient forces and biogenic pumping may also be involved.

Thus, endolithic organisms can enhance the environment they inhabit by removing various secondary coatings that cover such translucent minerals as quartz and feldspar. Mainly it relates to the iron films sometimes with admixture of OM. The bleached pattern is clearly a secondary feature on observed paragneisses and more rarely granitoids: it is patchy and adjacent to the iron stained spots, as well as associated with the areas of higher cryptoendolithic activity. Actually exfoliation is stimulated by formation of such bleached weathered morphones and microhorizons which lack the cementing iron oxides.

In both types of endolithic systems – cyanobacteria and lichen dominated – we found the most developed fractures network near the bleached iron depleted layers. We could clearly show that the “perforated” microcellular pattern was attributed to the feldspar zones (Fig. 1d) which points towards acidic weathering mechanisms originating both from cyanobacteria and fungi. At the same time cyanobacteria driven alkalization events occurring during photosynthetic activity were reflected in quartz degradation patterns. The weathering potential of cyanobacterial biofilms is really huge, as they can maintain completely different geochemical states in micrometer thick layers in contrast to neighboring microenvironments^30,31^. The same is true for lichens with powerful weathering abilities^32^ attributed to the mycobiont.

**Figure 3:**
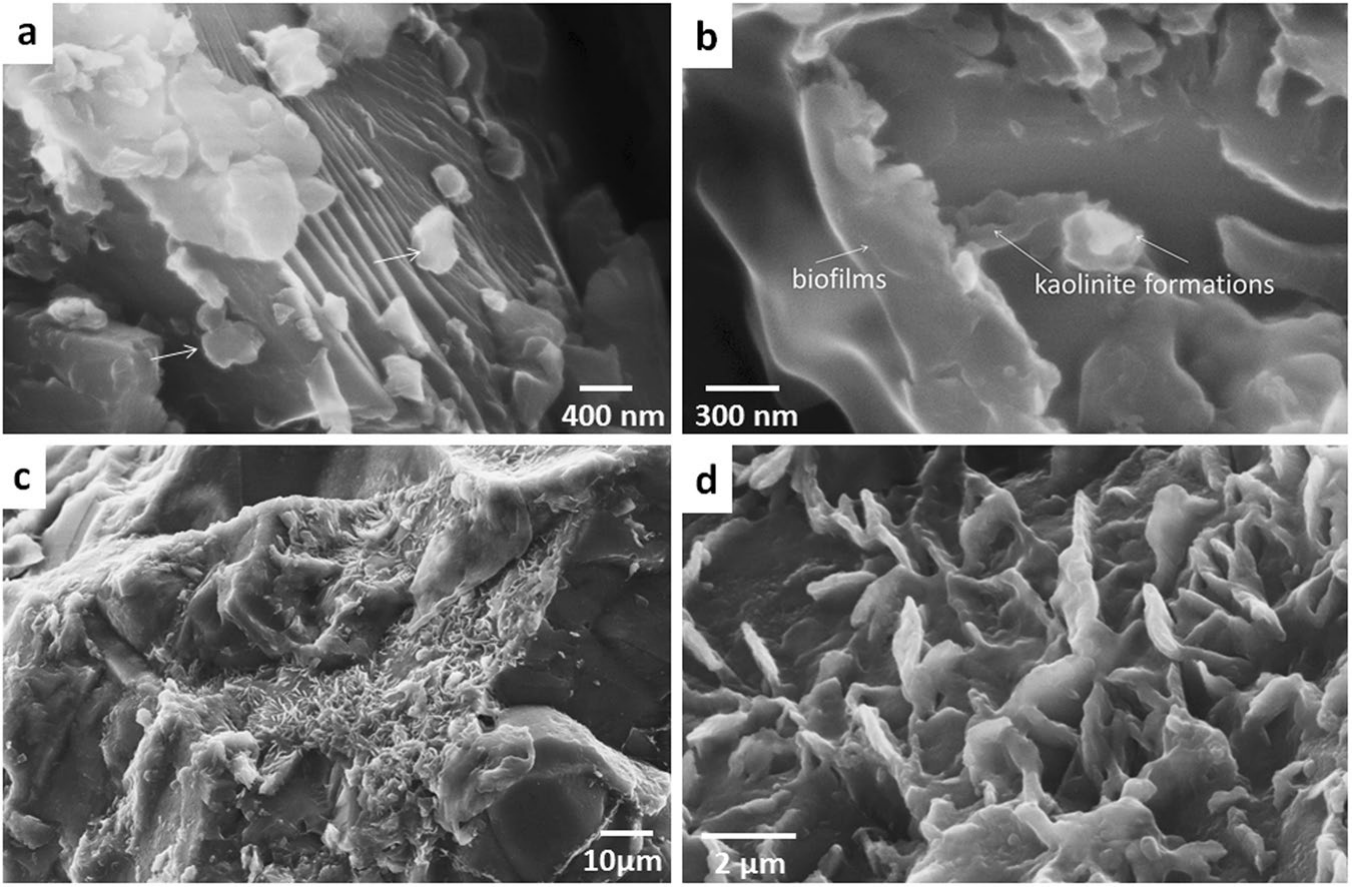
Clay minerals in endolithic organo-mineral horizon vs. deeper gneiss rock layer without biofilms: (**a**) – kaolinite particles and aggregates on feldspar grains; (**b**) – coalescence of organo-mineral films and kaolinite particles; (**c**,**d**) – minerals of smectite group in pores of initial rock (gneiss, Thala Hills, East Antarctica).

Clay minerals in endolithic systems do occur and regardless their genesis (biogenic or rock matrix inheritance) are expected to provide preferential spots for OM sorption as in common soils^33^. We observed different morphologies of clay minerals in SEM. Kaolinite formations of characteristic hexagonal shape were largely attributed to the endolithic organo-mineral horizons (formed in gneiss), more abundant on feldspar grains in comparison to other minerals (Fig. 3a), but closely associated with biofilms if those were present, and that was the most common case (Fig. 3b). Close occurrence of biofilms and clays could be partially due to good water storing capacity of the latter ones. The deeper layers (>5 cm) of gneiss had clean surfaces of primary minerals exposed, while clay minerals were rare, found in individual pores (Fig. 3c) and exhibited prominent morphology of smectite group species easily distinguished from other clay minerals (Fig. 3d). Kaolinite in comparison to smectite species are formed at different pH: smectite is mainly attributed to the alkaline environment, and kaolinite – to acidic. Alkaline conditions are often created during rock cata- and metagenesis. Acidic ones are normally found in subaerial lithobiontic environment, especially dominated by fungi and lichens (although for a very short period alkalization by cyanobacteria could occur). So we assume that there was a mediation of geochemical transformations by cryptoendolithic organisms resulted in kaolinite abundance in the thin suaberial layer when compared to the deeper lithomatrix. However, occurrence of kaolinite prior to cryptoendolithic colonization could not be completely excluded. Blackhurst *et al*.^34^ also found high kaolinite content in cryptoendolithic layer of Antarctic sandstone and proposed weathering *in situ* of feldspars to clays; he concluded that endolithic organisms are the architects of mineral weathering within the layers that they inhabit.

### Organic matter in endolithic systems

Endolithic biota comprised of microbial and cryptogamic photoautotrophs fuels rock interior with a variety of organic materials including polysaccharides, proteins, nucleic acids, lipids and others that can potentially be easily utilized with the help of heterotrophic components like bacteria and fungi. Estimates of the total OM contained in endolithic systems of the Antarctica Dry Valley rocks range from 7 to 177 g m^−2^ of the rock surface^35,36^. The average organic nitrogen content in the sandstone specimens from the Ross Desert^37^ varied from 1.4 to 6.0 g m^−2^.

**Figure 4:**
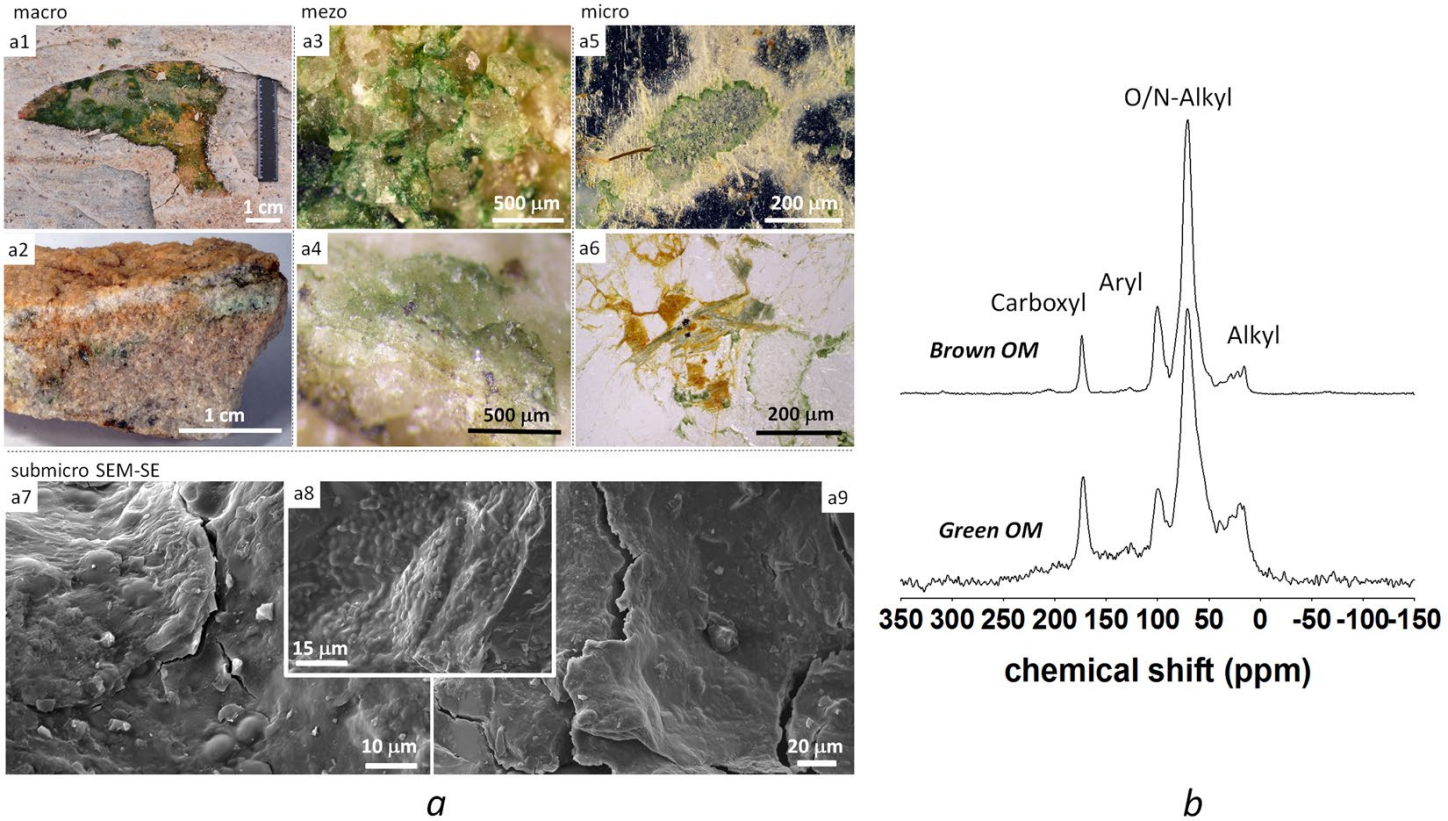
Organic matter at the different hierarchical levels of endolithic system (**a**) and its chemical composition (**b**): a1 – exfoliation reveals primary production of OM inside leucogranites at the Larsemann Hills of East Antarctica (photo in plan); a2 – vertical profile of endolithic system inside gneiss at the Thala Hills of East Antarctica with cyanobacteria and lichen dominated organic layers, bleached eluvial and illuvial horizons differentiated by Fe and C content; a3 – cyanobacteria biofilms covering quartz and feldspar grains; a4 – flake-like quartz degradation induced by cyanobacteria biofilm; a5 – weathering pattern on feldspar and quartz around the cyanobacterial biofilm and produced by it, reflected light image of the thin section in dark field, light areas indicate increased reflection of the incident light from highly weathered surfaces; a6 – thin section through cryptic organogenous horizon of endolithic system indicating biofilms penetrating between quarts and feldspar grains, several generations of OM are present including chlorophyll, carotenoids and Fe-containing organo-mineral coatings, reflected light image in bright field; a7,a8 – SEM micrographs (SE-secondary electrons) of cryptic fresh cyanobacterial coatings; a9 – SEM-SE micrographs of mature partly fossilized organo-mineral coatings without clear cellular morphologies. (**b**) - chemical composition of green and brown OM generations in endolithic system according to the ^13^C-CPMAS NMR, for integration, chemical shift regions were used as given: alkyl C ((−10) to 45 ppm), O/N-alkyl C (45 to 110 ppm), aryl/olefine C (110 to 160 ppm) and carbonyl/carboxyl/amide C (160 to 220 ppm).

The samples explored in this study had carbon content within 0.4–3.7%, nitrogen – 0.04–0.58%, C to N ratio – 8.7–12.5, and δ^13^C – within −22.5 to −24.2‰. Organic matter at the different hierarchical levels of endolithic system was represented mainly by various forms of biofilms and organo-mineral coatings (Fig. 4a).

According to Raman spectroscopy which was widely applied to identify *in situ* compounds in endolithic systems from extreme environments the individual molecules also comprise UV protectants (scytonemin, carotenoids), cryoprotectants (trehalose), antidesiccants (calcium oxalate monohydrate and dihydrate), chlorophyll and others^38,39^.

Upon senescence and microbial decomposition of degradable structures some refractory species could be accumulated in the rocks. Saiz-Jimenez^40^ summarized the data on more chemically stable compounds produced by lithobionts *in situ* which could include melanin widely present in endolithic systems and arguably considered as precursor of humic substances, sporopollenin like substances, various biopolymers of cyanobacterial origin highly resistant to drastic chemical treatments and consisting of long, unbranched saturated hydrocarbon chains, and others. Using ^13^C NMR Zelibor *et al*.^41^ found paraffinic carbon (most likely associated with cell walls) contributed to chemically refractory OM fraction derived from green algae cultures.

Here we present for the first time the chemical composition of OM from endolithic horizons in East Antarctica derived from ^13^C-CPMAS NMR spectroscopy. It revealed a clear dominance of O/N-alkyl C and, thus, the production of rather labile OM (Fig. 4b). The composition is comparable to the one from biological soil crusts in temperate regions dominated by polysaccharides, aliphatic biopolymers, and proteinaceous compounds as reported by Dümig *et al*.^42^. Interestingly the green forms in comparison to the brownish varieties of the endolithic biofilms showed substantially higher amounts of aliphatic and aromatic OC, which is believed to be more chemically stable. However, protection mechanisms of organisms themselves could be involved here, e.g. the storage of triacylglycerols in algae is assumed to be an adaptation to photo-oxidative and other environmental stress^43^.

### ^14^C activity of endolithic organic matter

Using ^14^C dating of the lipid fraction extracted from endolithic systems Bonani *et al*.^44^ assumed that microbial communities inhabiting rock fissure networks in the Ross Desert of Antarctica may belong to the oldest living organisms in existence on Earth. The reported calibrated ages of over 10^3^ years mainly result from very low rates of metabolism in this extremely inhospitable climatic conditions. Interestingly the authors interpreted these tremendous ages resulting from living organisms (cyanobacteria). But it can be assumed that parts of the aliphatic fraction are basically not the living biomass anymore but stabilized soil organic carbon in organo-mineral associations in the interior of these endolithic systems. Lipids derived from photosynthetic organisms can be preserved in sediments over geologic timescales and of all the classes of biomarkers studied to date they seem to be the most abundant in ancient organic matter^45^. We report that younger “ages” of endolithic systems are common (Table 1) decreasing to n*10^2^ years or having high values of fraction modern (F^14^C > 1), such younger “ages” are supported by recent data of Colesie *et al*.^36^. These values clearly represent the bulk organic carbon in the samples whereas the values reported by Bonani *et al*.^44^ may mainly represent a more stable aliphatic OM fraction, presumably of non-living OM in organo-mineral associations. The comparably high calibrated mean ages of the OM sampled from these endolithic microenvironements can be assumed to result from stabilization of OC in organo-mineral associations, but also as a function of the extreme environment, noting that the rates of decomposition are very slow and primary produced OM could possibly stay as it is for a very long time.

**Table 1:** ^14^C activity of endolithic organo-mineral horizon (according to our previous studies^22,63^ and unpublished data).

Sample	F^14^C	^14^C yr BP (1σ)
Thala Hills Oasis, granite-orthogneiss	1.084 ± 0.003	—
Thala Hills Oasis, pegmatite	1.062 ± 0.003	—
Thala Hills Oasis, orthogneiss	0.980 ± 0.003	160 ± 25
Thala Hills Oasis, granite	0.976 ± 0.003	190 ± 25
Larsemann Hills, granitoid, vertical surface	1.029 ± 0.003	—
Larsemann Hills, granitoid, horizontal surface	0.942 ± 0.003	480 ± 25
Aerodromnaya Hill nunatak, near Schirmacher, granite-gneiss	1.070 ± 0.003	—
Aerodromnaya Hill nunatak, near Schirmacher, granite-gneiss	—	1780 ± 30

### Organo-mineral associations as evidenced by NanoSIMS and SEM-EDX

By using NanoSIMS we were able to explore the elemental distribution directly at the surface (~10 nm depth resolution) of biofilm-to-mineral biogeochemical interfaces.

**Figure 5:**
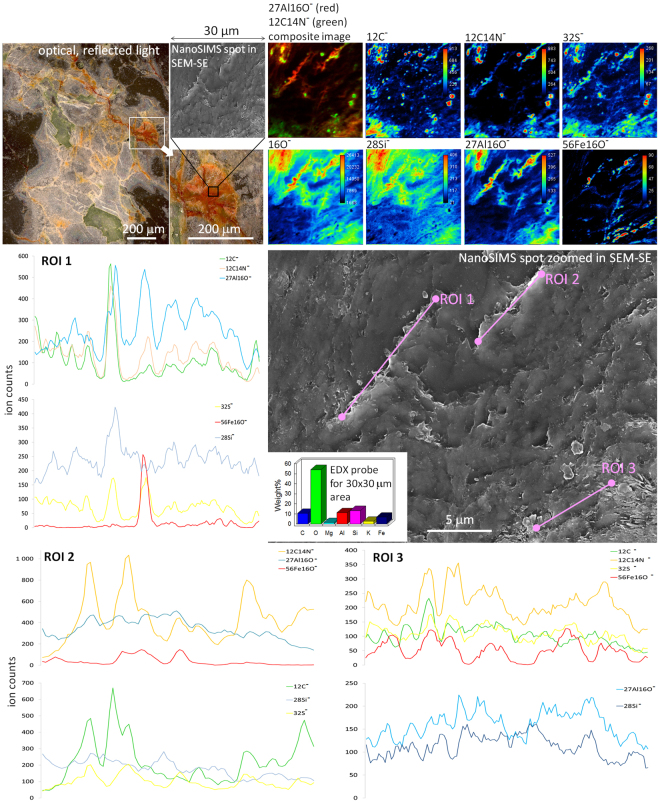
NanoSIMS study of the 30 × 30 μm site at the surface of organo-mineral association in combination with SEM-SE/EDX and optical microscopy in reflected light. ROIs – selected regions of interest with different correlations between ions. Optical microscopy initially suggested close links between OM and Fe species. Nitrogen rich OM (higher ^12^C^14^N^−^ counts) is more frequently directly colocalized with other mineral compounds, mainly containing aluminum (seen at the NanoSIMS ^27^Al^16^O^−^/^12^C^14^N^−^ composite image and ROI 1 ion spectra). Rough and flaky patterns seen in SEM-SE in vicinities of ROIs 1–3 are indicative of clay particles, and these regions are enriched in ^12^C^14^N^−^ and ^32^S^−^.

**Figure 6:**
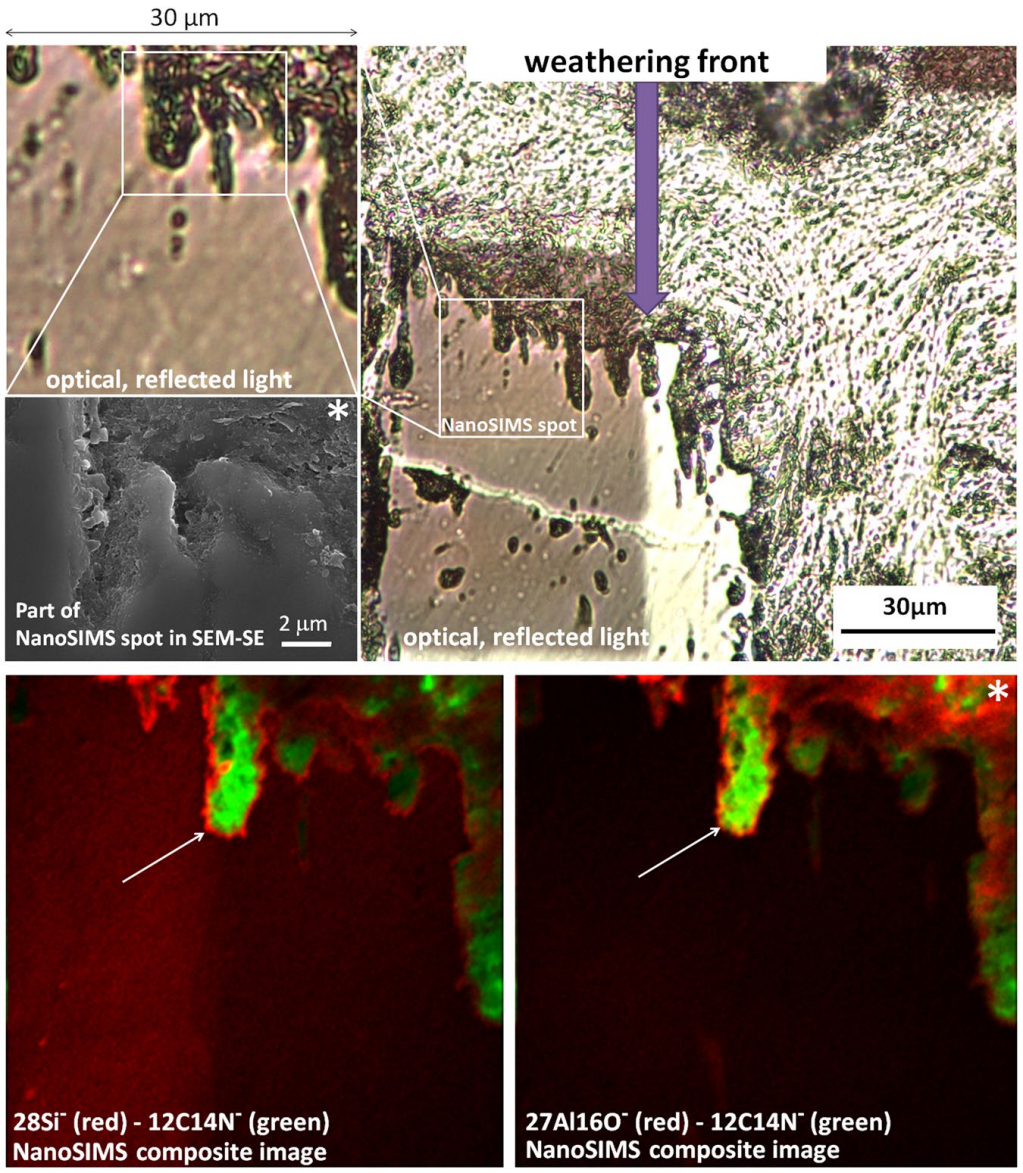
Endolithic weathering front induced by cyanobacteria-to-mineral interactions. Cryptoendolithic cyanobacteria which originally occupy large pores in the rock (seen in the right and upper part of the optical images as green cellular pattern) enhance their habitat by dissolution of surrounding minerals (employ locally euendolithic strategy). Increased ^28^Si^−^ and ^27^Al^16^O^−^ ion counts right at the forefront of weathering (white arrows) suggest the cyanobacteria-to-feldspar biogeochemical interfaces in the etching pits. These zones demonstrate amorphization of aluminosilicates with higher ion counts in the newly formed amorphous areas in comparison to the crystalline structures of primary minerals. Also right upper part of NanoSIMS and SEM-SE images suggest close association of clays and organic matter rich in nitrogen (area in vicinities of asterisk).

The studied elements detected as ^12^C^−^, ^12^C^14^N^−^, ^16^O^−^, ^28^Si^−^, ^32^S^−^, ^27^Al^16^O^−^, ^56^Fe^16^O^−^ secondary ions exhibited sophisticated spatial patterns at submicron scale (Figs 5, 6 and Supplementary Figs S2–5). The higher lateral resolution of NanoSIMS in contrast to SEM-EDX allowed to demonstrate that at some spots OM is interlayered, encapsulated and occluded in the mineral components, which seems close to taphonomic processes. However, there are numerous spots where OM derived secondary ions (^12^C^−^ and ^12^C^14^N^−^) are distinctly colocalized with mineral components indicating the prevalence of sorption mechanisms and the development of organo-mineral associations. We can clearly emphasize an increased role of aluminum rich moieties (e.g. clay minerals) over silicon ones in sorption comparable to what is known for clay-OM complexes in common soils (for instance OC patches on soil microaggregates^33^). As follows from our data set the microscale spots of organo-mineral associations are confined predominantly to the regions of extracellular polymeric substances (EPS) occurrence rather than to the areas with pronounced cellular morphologies. By high resolution imaging we are able to demonstrate the preferential OM occurrence in micron size defects on mineral surfaces, larger etching pits and attributed to clay mineral morphologies, yet not fully covered by OM. It was shown before that organo-mineral associations are preferentially located at edges and rough surfaces of clays which act as nuclei for OM attachment^33^. This will subsequently foster OM-to-OM selective sorption and thus the growth of OM coverage. Additionally, we found that nitrogen rich OM (higher ^12^C^14^N^−^ counts) is more frequently directly colocalized with mineral compounds, mainly aluminum (Fig. 5). This is consistent with the “onion” layering model of OM accumulation in common soils^46^ in which N-containing compounds are preferentially adsorbed onto mineral surfaces. Based on correlation pattern (scatter plots, Supplementary Fig. S6) between the two OM derived secondary ions (^12^C^−^ and ^12^C^14^N^−^) we conclude that several different OM generations are present in parallel in the endolithic systems. We also could show a very close correlation between sulfur rich compounds (^32^S^−^ secondary ion) with ^12^C^−^ and ^12^C^14^N^−^ indicative of presumably proteinaceous compounds. Only a limited number of spots showed a colocalization of iron (^56^Fe^16^O^−^) and OM pointing to a lower importance of iron oxides for OM storage in the endolithic systems.

We found increased ^28^Si^−^ and ^27^Al^16^O^−^ secondary ion counts (Fig. 6) right at the forefront of weathering, the cyanobacteria-to-feldspar biogeochemical interfaces in etching pits. We assume that these zones demonstrate the amorphization front in aluminosilicates with higher ionization rates in the newly formed amorphous areas in comparison to the crystalline structures of primary minerals. Silanol-siloxane transitions and the formation of allophane/(proto)imogilite cannot be excluded here. Such biogeochemical interfaces were reported by Benzerara *et al*.^47^. By using STXM-NEXAFS at the Al K-edge and TEM the authors were able to confirm disordered aluminosilicate, such as allophane, at the microorganism-to-mineral interface in Fe-Mg-orthopyroxene, with an amorphous Al-rich layer at the nanometer scale like presented here.

These mineral transformation enhancements by biota suggest that the same organisms (cyanobacteria) originally occupying cryptoendolithic niche can employ also an euendolithic strategy, penetrating deeper in the rock from the crypto habitat. Although for phototrophs penetration is very limited in space. Endolithic cyanobacteria can differentiate mechanisms of weathering^30,31^ by dramatic but short-time pH increase up to 9 during photosynthesis (conductive for quartz weathering) and significant pH decrease <4 (conductive for feldspar weathering).

### Endolithic habitat and inhabitant interrelation

Specifically for cryptoendolithic communities the pre-existence of a weathering structure and/or porous space is very important. The rock is often treated by the processes of physical weathering granting the fissure networks and pores for organisms to settle. But once in the rock organisms start to enhance and alter it and also produce the new organo-mineral products (organo-mineral coatings as seen in SEM-EDX and NanoSIMS) inside the rock. As a result initial lithomatrix is transformed *in situ* by biogenic and abiogenic factors, the products of transformation are retained, migrate and/or removed, the vertical heterogeneity is established in a form of microhorizons composing microprofile resembling soil sequences. A wide range of endolithic bioweathering products as we showed here and also previously^18^ includes the fine earth, abundant organo-mineral films, carbonates both on an exterior of cyanobacterial biofilms and within EPS, and oxalates. From our point of view, these newly formed substances can be considered as *in situ* microproducts of the endolithic pedogenesis.

We assume that endolithic colonization is not necessarily just an escape from harsh conditions or a secondary adaptation, it could also be taking up the space available. At least that is what we learn from modern analogues: endoliths can be found in a wide range of conditions from polar deserts to subtropics and tropics. In milder climatic conditions the endolithic form of life often goes together with the epilithic one and moreover is a neighbor to the lush vascular vegetation. So the endolithic niche is colonized in many climatic conditions, but this process strongly depends on the physical properties of the habitat, lithological variables, e.g. the presence of translucent minerals, their size, cement between primary mineral grains and of course sufficient porous space. If the rock is not favorable for certain type of endoliths (e.g. like many basalts for cryptoendoliths) it will not be colonized (or intensively colonized) in this specific niche even in harsh conditions. In extreme environments like oases of East Antarctica (on favorable rocks) endolithic niche is sometimes just the only one left to be securely occupied by photoautotrophs, and, thus, so visible in comparison to other environments. Thus, habitat goes ahead of inhabitance, but once the niche is colonized it is processed and altered by organisms. At this stage the inhabitants change the habitat. There are no significant obstacles to assume that exposed rocks in Precambrian like granitoids, some gneisses and sandstones had porous space and of course they had such translucent minerals as quartz and feldspars, thus they could be colonized by biota when conditions became favorable. Afterwards organisms could have changed the surrounding subaerial segment of the rocks. At some point it could be adaptation, but more ecological than physiological to escape for instance high UV levels.

### Scarce geological record of endolithic systems and their possible signatures

Understanding of weathering patterns produced in modern endolithic systems of extreme environments which lack the noise from vascular vegetation may help to better search for traces of endolithic activity in the distant past. However, it is not just subaerial endolithic life but generally microbial terrestrial life in Precambrian that has very poor geological record, especially in comparison to the deep aquatic or even shallow-water settings. The record of the past is obviously better preserved in sedimentary basins of various scales and we get only few and often dispersed signals about vast arid, mountains and rocky regions that could have existed in Precambrian as well as their landforms and critical zone nature and thickness. Poor preservation in the geological record does not directly mean that terrestrial life did not exist on the early continents, but could lead to biases in the interpretation of terrestrial paleoenvironments^48^. This also relates to our knowledge on endolithic colonization of early landmasses. It was proposed^49^ that the earliest oxidative-weathering reactions occurred in soils and benthic environments on land before the Great Oxidation Event. Such settings possibly included rather arid areas with biological soil crusts and riverbed, lacustrine, and estuarine sediments with freshwater microbial mats. Ancient cratons that preserve signals of oxidative weathering have stabilized ~3.0–2.5 Ga^49^ and the evolution of landmasses together with its microbial colonization perhaps contributed significantly to the manifestation of oxidative weathering. It is not possible yet to draw a reliable lower time boundary for the start of land colonization by organisms in general, since it depends largely on calibration by fossil findings, which are still few. Thus, the same is true for terrestrial microbial photoautotrophs including those which possibly occupied endolithic niches. There is geochemical evidence for terrestrial ecosystems of prokaryotic origin as early as 2.8–2.6 Ga^5,50^. The molecular clock estimates for land colonization by prokaryotes refer to the interval between 3.1–2.8 Ga and the antiquity of cyanobacteria, which play nowadays a major role in endolithic systems, is also close to 3.0 Ga^51,52^. Morphological features of stromatolites point (although often just putatively) to the presence of cyanobacteria by 3.5–3.4 Ga^53^. But this refers mainly to aqueous sedimentary environment. Microbially induced sedimentary structures (MISS) associated with biomats in siliciclastic rocks date back to 3.2–2.9 Ga^54,55^. They are primarily related to shallow-water marine, tidal/intertidal and coastal environments and are regarded as the settings with periodic subaerial exposure and important pathways for a biological transition from water to land^48^. Recently findings by Homann^55^ extended the record of Archaean 3.2 Gy-old microbial ecosystems to the near-coastal but clearly terrestrial settings with temporary subaerial exposure. Discovery of microbial communities in the Paleoarchaean terrestrial habitats indicates their potential for adaptability and resilience in colonization of the new ecological niches on land^55^. Thus, one view is that this process was connected with evolutionary leap^55^ in adaptation to the new harsh environments on land such as desiccation, erosion, elevated temperatures and UV radiation. But probably the endolithic niche could mitigate many of these factors like it happens today without need for significant evolutionary changes, e.g. obligatory obtaining of the photoprotective pigments. Such organisms as cyanobacteria could have migrated from aqueous environments through wet coastal, floodplain, estuary pathways or through airborne dispersal to the moisture supplied subaerial segments of rocks. The model by Cockell & Raven^56^ suggests that even under a harmful UV radiation environment assumed on the Archaean Earth, there would be sheltered habitats suitable for primitive photosynthetic organisms. There are fossil data^57^ allowing to draw an assumption that the microhabitats within vesicles of pillow basalt could have provided UV shelters for microbial cryptoendoliths of (chemo)lithoautotrophic origin on early Earth. However, these findings relate to deep aqueous conditions, as well as other discoveries of microscopic borings in basaltic glass attributed to ancient euendolithic bacteria^17^. As mentioned by McLoughlin^17^, the conservation potential of euendoliths is possibly higher than of crypto- and chasmoendoliths, and we believe the same is true for the subaerial endoliths.

Eukariotic components as green algae, fungi and lichens are also very important for modern endolithic systems. Unambiguous record of eukaryotic microfossils dates back to ∼1.8 Ga and calibrated molecular clock points toward similar ages of 1.87–1.68 Ga^58^, recent findings^59^ proposed 2.8–2.7 Ga-old tabular microfossils from a terrestrial lacustrine environment similar to modern siphonous microalgae.

**Figure 7:**
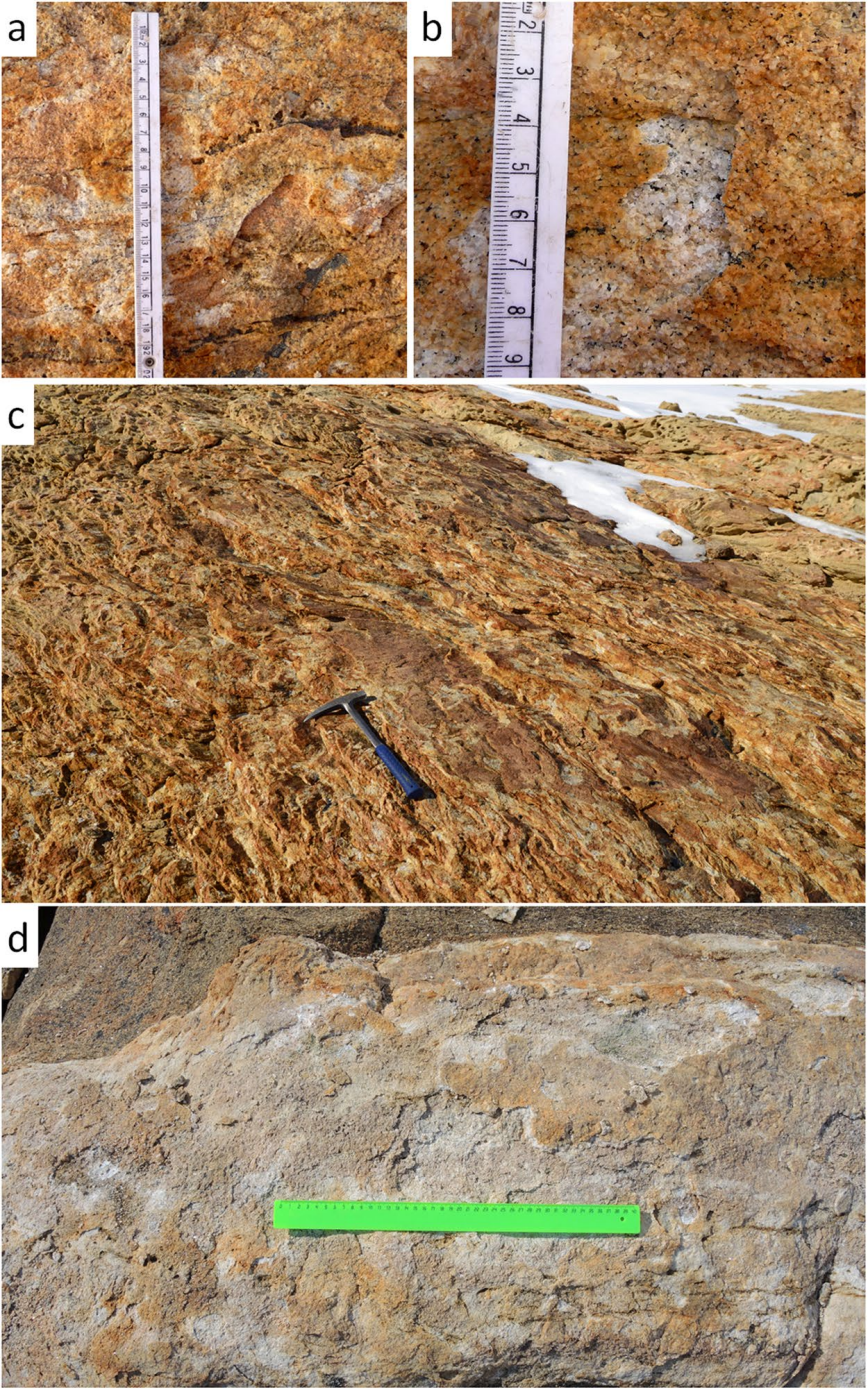
Bleached and iron stained patterns on paragneiss at the Larsemann Hills of East Antarctica associated with cryptoendoliths: (**a**,**b**) – bleached spots are located in microdepressions indicating relatively recent exfoliation; (**c**,**d**) – bleached and stained patterns are extensively reproduced in a rocky landscape. These trace fossils could be the keys for search of ancient subaerial endolithic systems in the geological history of Earth.

Search for the signs of ancient subaerial endolithic systems is a challenging future goal that we try to evoke by this study. Cryptoendolithic microbes of modern Antarctica can leave detectable fossils^16^, biomarkers and inorganic traces. Some of the signatures of endolithic systems that have potential for long-term preservation are reported in our study and comprise the bleached eluvial horizons (Fig. 7) also noted by Friedmann and Weed^60^ as possible endolithic trace fossils, and weathering patterns as revealed by SEM-EDX and NanoSIMS. However the latter should be accurately interpreted as similar signatures could be produced by the same organisms, but in different ecological niches.

### Life as a fatal process for endolithic systems: addressing inconsistency between scale of biofilms and paleosols

Although there are various pathways for OM to be stabilized in endolithic systems for time scales of n*10^3–4^ yr or even longer the life of these soloids is often interrupted earlier, within <n*10^2–3^ yr. Subaerial weathering and endolithic pedogenesis gradually make bonds between the rock minerals very loose and finally destroy the system through exfoliation. Organo-mineral products of endolithic origin are eroded and distributed along the landscape, so not just trace fossils left in place on the rock as described above, but also biomarkers and biosignatures accumulate in the surrounding sedimentary environment. This process was defined earlier as a self-destroying pedogenesis^22^ suggesting that endolithic systems never develop to a full-scale soil *in situ* but contribute to the soil formation along the landscape. But after the system is eliminated the subaerial weathering and colonization continue; endolithic pedogenesis starts over again.

This knowledge might help to address the paradox of inconsistency between thin biofilms or biocrusts which are believed to comprise biogenic covers on rocks in Precambrian and thick paleosol profiles of that time, similar in many ways to deeply weathered soils at the land surface today^1,61^. How could this happen? Quite possibly these were the abiotic agents (e.g. carbonic acid, hydrothermal fluids) that deeply processed soils, and biota driven alterations were confined mainly to the top part. However, *in situ* interactions between agents of prokaryotic biosphere and minerals cannot be excluded in deeper subsoil. Interaction could have happened over a large time scale to produce deep bioweathered profiles. We speculate here that some contribution into thick paleosol bodies may originate also from long-term replenishing of soils in sedimentary positions by eroded decay products of hard rocks being processed by biofilms including endolithic ones, thus containing their remnants. Such soil build up is happening now for instance in Antarctic accumulative landscapes as evidenced from isotopic data. The Dry Valleys soils have clear isotopic signature (δ^13^C and δ^15^N) of endolithic organisms^23^, thus meaning they were partially formed from the fine earth produced through (crypto)endolithic pedogenesis. This could also relate to the emergence of biogeomorphological forces on Earth^62^ which acted through endolithically induced regular exfoliation and reshaped mineral surfaces and landforms^31^.

## Conclusion

**Figure 8:**
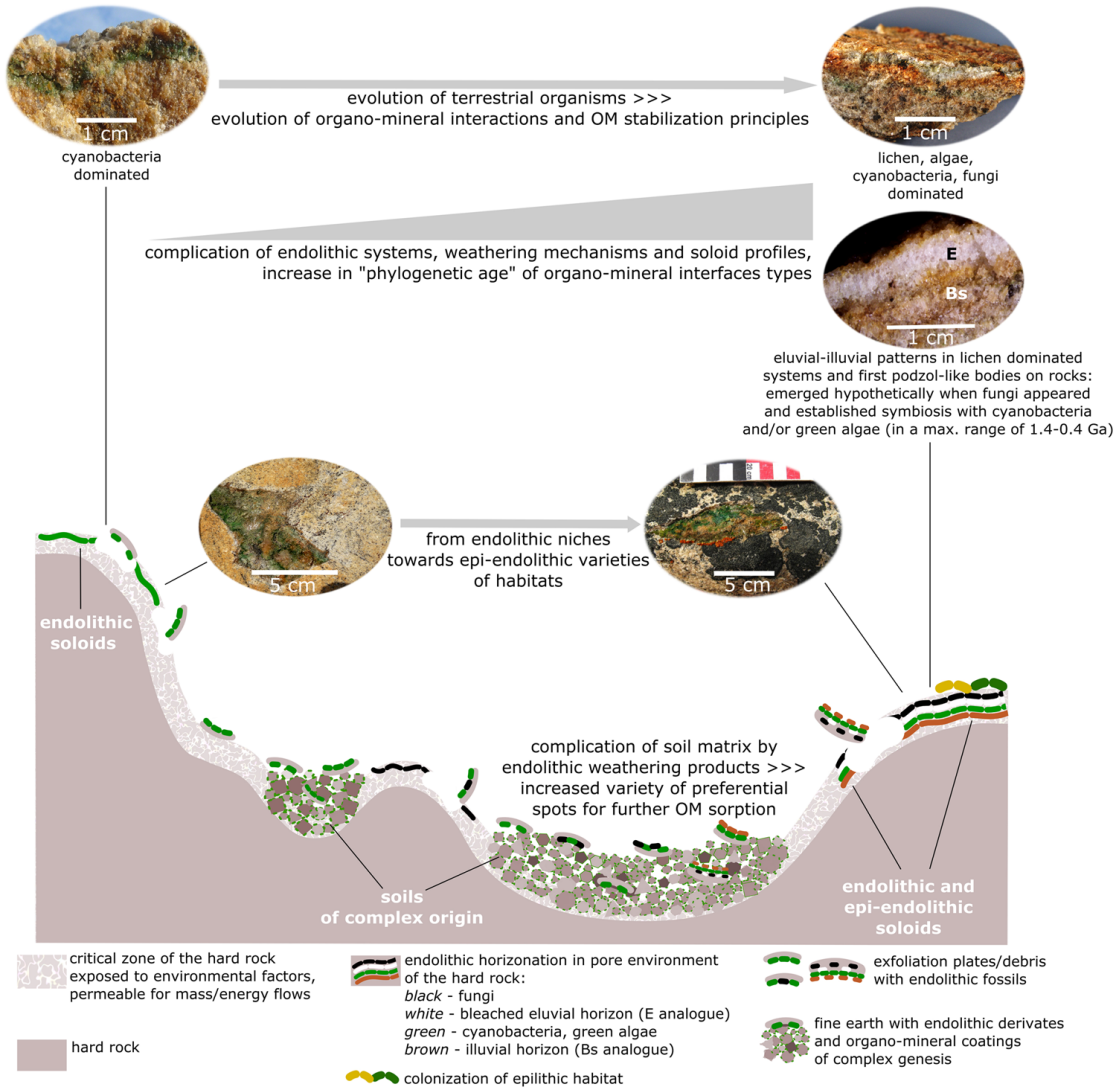
Evolution of subaerial endolithic systems (soloids) and their contribution to the increased complexity of landscapes. Here we refer mainly to cryptoendolithic varieties. Lower estimate of time boundary (1.4 Ga) for fungi appearance in endolithic systems is proposed upon protein sequence data by Heckman *et al*.^7^; upper boundary of 0.4 Ga stands for various unambiguous fossil data on fungi and lichens in cherts.

Using an actualistic approach we demonstrate that transformation of silicate rocks by endolithic organisms is one of the possible pathways for the beginning of soils on Earth. This process (1) led to the formation of soil-like bodies on rocks *in situ* (soloids) and (2) contributed to the raise of complexity in subaerial geosystems through fine earth production, provision of preferential spots for further OM sorption and, thus, the soil cover build up (Fig. 8).

Subaerial endolithic systems leave various organic and inorganic signatures in the landscape. The bleached & stained patterns are among the most prominent trace fossils. This could be the key to decrypt the record of the ancient endolithic systems on Earth.

We observed surprisingly diverse types of organo-mineral interactions and potential pathways for OM stabilization in the studied endolithic systems from East Antarctica. This emerging knowledge corresponds well to the modern concepts of organo-mineral interactions in common soils. Relatively simple systems of microbial and/or cryptogamic origin that exist and replicate on Earth over geological time scales demonstrate the principles of OM stabilization strikingly similar to those known for modern full-scale soils of various climates: 1) physical protection through occlusion/encapsulation in soil aggregates, 2) sorption on clay minerals and amorphous phases, formation of simple organo-mineral associations, 3) environmental protection through hydro-thermal barriers and 4) formation of more chemically refractory compounds, including those produced for self-protection purposes. The data presented indicate that the set of early principles of organo-mineral interactions and OM stabilization, which evolved in a certain manner according to the mineral and biological variables, has not changed fundamentally with the advent of vascular plants. However, this should not be confused with a set of biopolymers, their derivates and decay products, which became more and more sophisticated with the evolution of live on Earth. What have changed are the ratios and intensities of contribution between fundamental mechanisms of organo-mineral interactions. Thus, the mechanisms of soil formation and OM stabilization well known today may date back to the “first joint steps of biota and soils” on land.

## Methods

### Sampling design

Endolithic systems comprise endolithic organisms (cyanobacteria, chlorophyta, fungi, heterotrophic bacteria, lichens, etc.), mineral environment they inhabit and *in situ* altered organo-mineral byproducts. For the purposes of current research we worked with cryptoendolithic systems occupying empty spaces or pores inside a rock, further referred as simply endolithic. Although organisms inhabiting the cryptoendolithic niche can also locally employ an euendolithic strategy and promote weathering of surrounding minerals. Endolithic system is a worldwide phenomenon (Supplementary Fig. S1), however only East Antarctica provides the most “clean” environment lacking completely the influence from vascular plants and minimizing effects from epilithic and epi-edaphic biological covers. The samples of endolithic systems were collected from the exposed bedrock surfaces in coastal oases of East Antarctica: the Larsemann Hills (S69°20′, E76°20′), Thala Hills (S67°40′, E45°20′), Schirmacher (S70°45′, E11°37′) and Aerodromnaya Hill nunatak (S70°47′, E11°37′). The bedrock in the Larsemann Hills consists of granitoids and gneisses with high content of translucent minerals like quartz and feldspars and additions of garnet and biotite. The most pronounced endolithic systems were expectedly found on leucogranites. Endolithic systems from Thala Hills were sampled from orthogneisses and enderbites containing feldspars, quartz, hypersthene, diopside, amphiboles (hornblende), and biotite. Gneisses with higher content of quartz/feldspars were chosen in Schirmacher oasis and neighboring Aerodromnaya Hill nunatak. Microclimatic conditions on these rocks are discussed in Mergelov *et al*.^22^. Two types of sampling designs were employed: boring of micromonoliths (0.7, 1.5 and 20.0 cm^3^ in volume) and multicomponent *in situ* excavation including (1) exfoliation plates with endolithic communities on the bottom sides and rock varnish/silica glaze on the upper sides, (2) endolithic organo-mineral horizon under exfoliation plates comprising mineral fine earth and biomass of the endolithic organisms, and (3) underlying rock without macro signs of organisms. Totally 20 systems were sampled.

Morphology of endolithic systems was studied on different hierarchical levels - under Leica MZ6 binocular, Carl Zeiss Axio Scope A1 and Nikon Eclipse E200 optical microscopes all with digital cameras. The samples were observed both in native state and in standard thin sections. Transmitted and reflected light, bright and dark fields were used to obtain maximum information.

X-ray microtomography (µCT) at Bruker SkyScan 1172 (Dokuchaev Soil Science Institute, Moscow, Russia) was used to study internal structure and fracture network in rock micromonoliths with endolithic systems. Application of µCT provided opportunity to visualize simultaneously the three major structural components of endolithic systems in intact samples: arrangement of mineral grains distinguishable in composition due to characteristic densities in X-rays, pore spaces in-between and weathering zones with biofilms and organo-mineral coatings/filaments.

Scanning electron microscopy (SEM) was performed in secondary electrons (SE) and backscattered electrons (BSE) modes at JEOL JSM-6610LV (Institute of Geography RAS, Moscow, Russia) with energy-dispersive X-ray spectroscopy (EDX by Oxford Instruments) employed for elemental analyses of native particles and polished resin fixed thin sections. SEM-EDX mapping of particles and thin sections were also used at the same spots as for NanoSIMS studies.

Nanoscale secondary ion mass spectrometry (NanoSIMS) studies were conducted at the Cameca NanoSIMS 50 L (Gennevilliers, France) of the Lehrstuhl für Bodenkunde, TU München, Germany. Electron multiplier secondary ion collectors were used for ^12^C^−^, ^16^O^−^, ^12^C^14^N^−^, ^28^Si^−^, ^31^P^−^, ^32^S^−^, ^27^Al^16^O^−^ and ^56^Fe^16^O^−^. Charging was compensated by an electron beam generated by the electron flood gun of the NanoSIMS. Samples were produced in a form of thin sections documented by optical microscopy and SEM before and after NanoSIMS measurements. Samples were coated by gold. Before analysis, impurities and the coating layer were sputtered away by using a high primary beam current. By combination of NanoSIMS and SEM-EDX measurements at the same spots we compared the completeness and quality of data received. Prior to the study reported here the subaerial endolithic organo-mineral coatings in East Antarctica were most extensively studied by means of SEM/TEM with EDX extension, which normally register emission of characteristic X-rays from a depth of ~1–3 microns, or more precisely from 1–3 cubic microns, depending on the electron beam energy. Although EDX probe is a powerful and indispensable tool to study organo-mineral interfaces, the data received by it may mislead (in comparison to NanoSIMS) upon various elements colocalization providing the average signal from submicron clusters, besides the tool is less capable than NanoSIMS in distinguishing lighter elements (e.g. C, N), especially at a high spatial resolution.

### Chemical analyses

The carbon and nitrogen contents in the endolithic organo-mineral horizon were determined by dry combustion of dried and finely ground samples at Elementar Vario ISOTOPE Cube in CHNS-analyzer mode. For the chemical analyses using ^13^C-CPMAS NMR spectroscopy (Bruker DSX 200 spectrometer; Bruker BioSpin GmbH, Karlsruhe, Germany) the crust material was scraped from the rock and finely ground. Samples were filled into zirconium dioxide rotors and spun in a magic angle spinning probe at a rotation speed of 6.8 kHz to minimize chemical anisotropy. A ramped 1 H pulse was used during a contact time of 1 ms to prevent Hartmann–Hahn mismatches. Chemical shifts are referenced to tetramethylsilane (TMS = 0 ppm). For integration, chemical shift regions were used as given: alkyl C ((−10) to 45 ppm), O/N-alkyl C (45 to 110 ppm), aryl/olefine C (110 to 160 ppm) and carbonyl/carboxyl/amide C (160 to 220 ppm).

### Accelerator Mass Spectrometry Radiocarbon Dating (^14^C AMS)

Sample preparation procedures that we applied to date organic matter derived from the endolithic organo-mineral horizons are described in details in Zazovskaya *et al*.^63^. ^14^C AMS measurements were implemented at the Center for Applied Isotope Studies, University of Georgia, USA.

### Data availability

The authors declare that the data supporting the findings of this study are available in the article and its supplementary part. Additional datasets generated and/or analysed during the current study are available from the corresponding author on reasonable request.

## Electronic supplementary material

Supplementary information
