# Supplementary material for: Alteration of rocks by endolithic organisms is one of the pathways for the beginning of soils on Earth
Source: Sci Rep. 2018 Feb 20;8:3367. doi: 10.1038/s41598-018-21682-6 (PMC5820250; doi:10.1038/s41598-018-21682-6)
Supplement: Supplementary file 1 — Supplementary information [file 41598_2018_21682_MOESM1_ESM.pdf]

Supplementary information

Article title: Alteration of rocks by endolithic organisms is one of the pathways for the beginning of soils on Earth

Authors: Nikita Mergelov, Carsten W. Mueller, Isabel Prater, Ilya Shorkunov, Andrey Dolgikh, Elya Zazovskaya, Vasily Shishkov, Victoria Krupskaya, Konstantin Abrosimov, Alexander Cherkinsky, Sergey Goryachkin

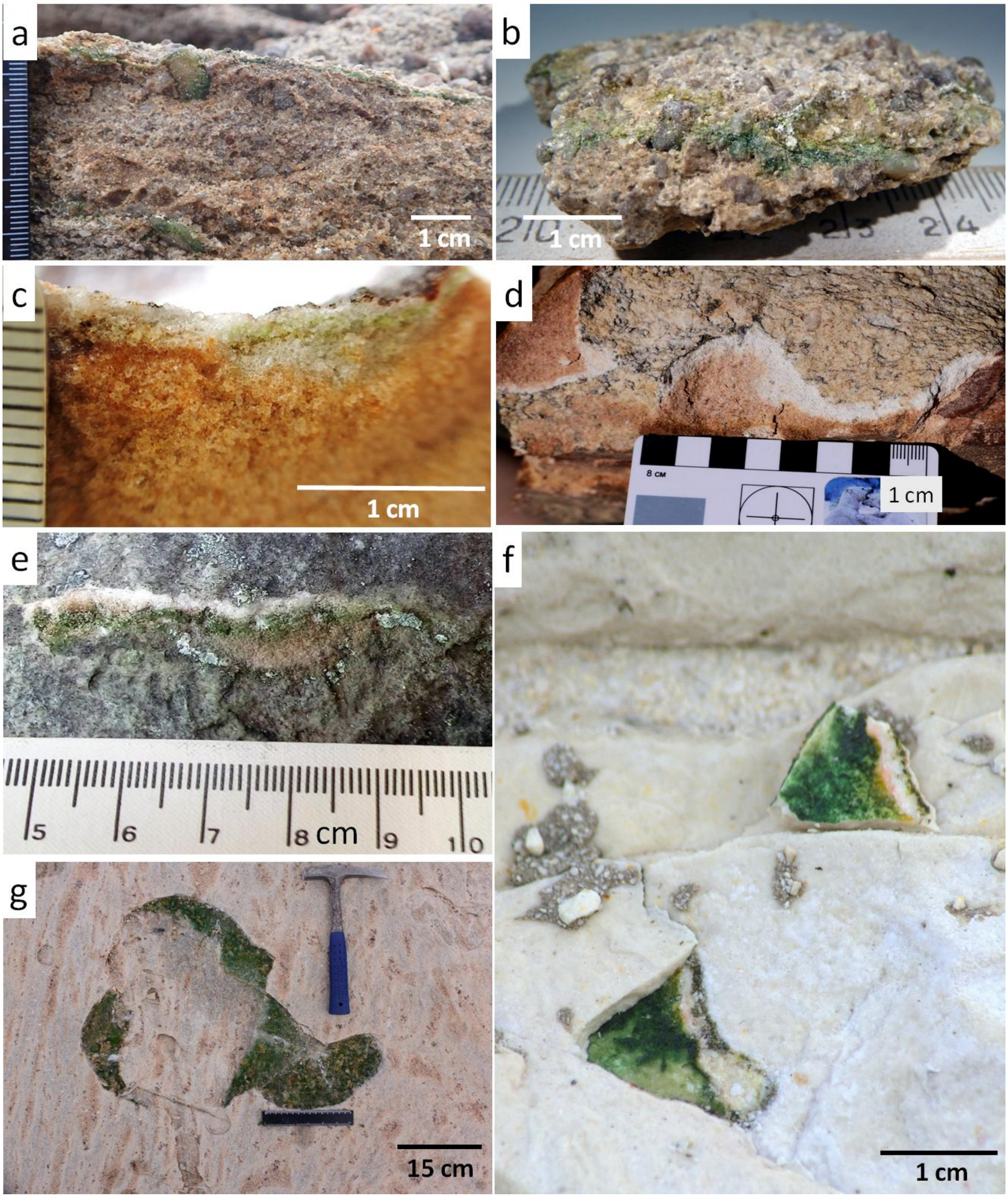

Figure S1. Endolithic system is a worldwide phenomenon, however only East Antarctica provides the most "clean" environment lacking completely the influence from vascular plants and minimizing effects from epilithic and epi-edaphic biological covers.
a, *b* – Cape Zhelanya, Novaya Zemlya High Arctic archipelago, conglomerates with increased quartz content; *c* – Blyde River canyon, South Africa, quartzite sandstones; *d* – Cape Fold Mountains, South Africa, quartzite sandstones; *e* – Plateau Ozark, Missouri, quartzite sandstones; *f* – Bari, South Italy, carbonate rocks; *g* – Larsemann Hills, East Antarctica, leucogranite with high quartz content.
We refer here to the cryptoendolithic varieties. South Africa and Missouri varieties combine both endolithic and epilithic forms of lichen colonization.

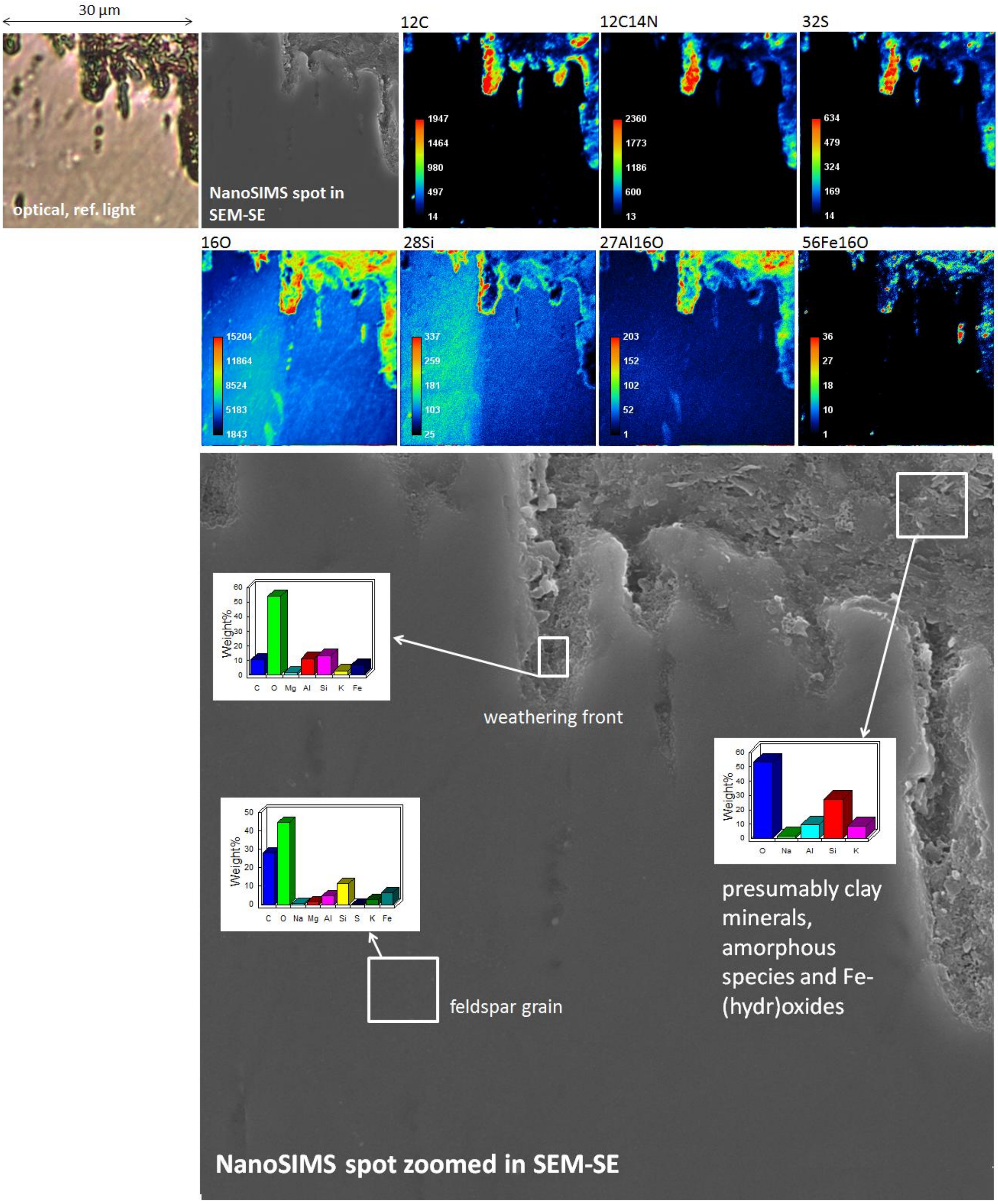

Figure S2. Weathering seen at the forefront of cyanobacteria-to-feldspar interaction (NanoSIMS data)

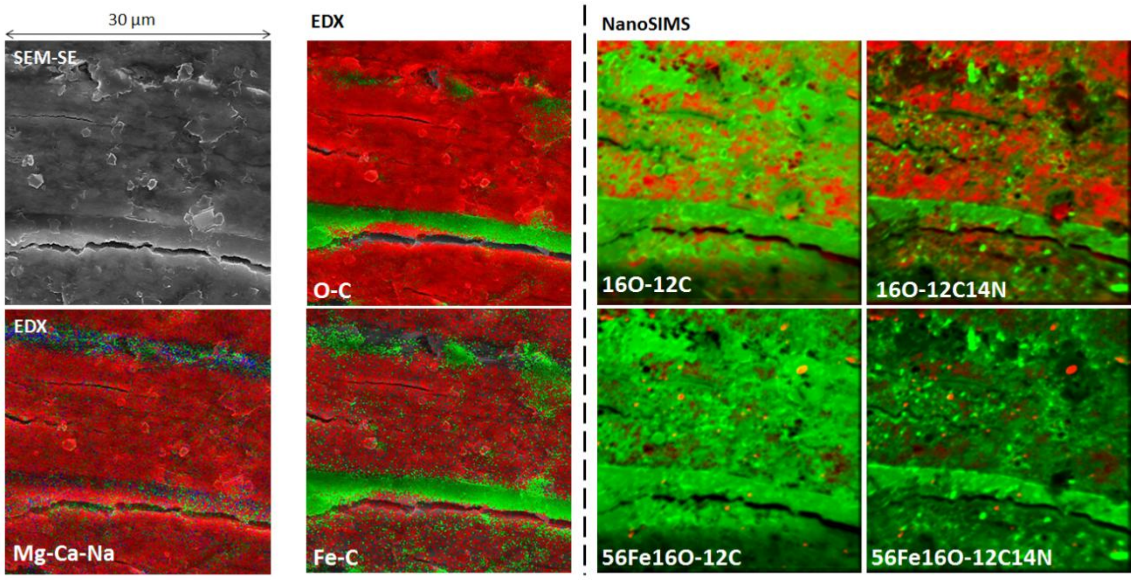

Figure S3. Comparison of EDX and NanoSIMS elemental distribution in composites (cyanobacterial biofilms interlayered with biotite flakes).
RGB sequence of colors: red matches the first element, green – second, blue – third.

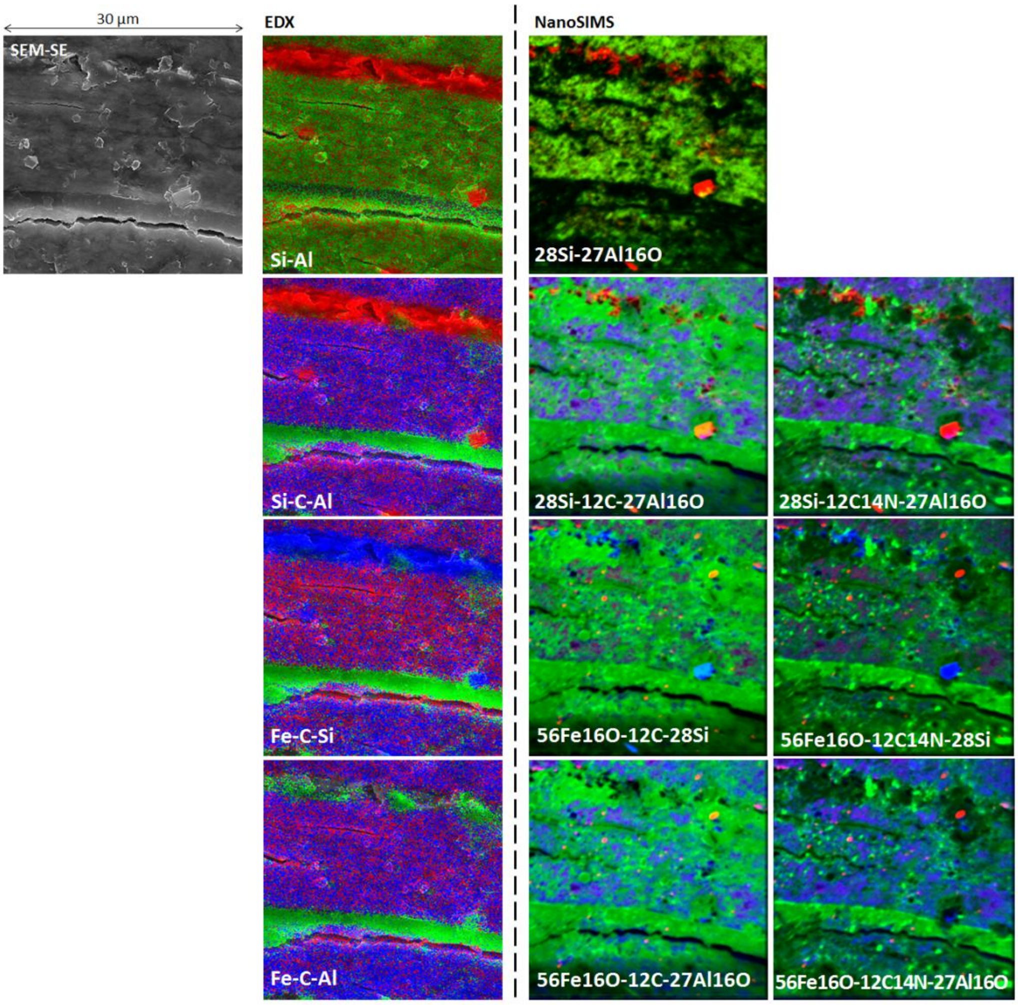

Figure S3-1. Comparison of SEM-EDX and NanoSIMS elemental distribution data in composite images (biofilms interlayered with biotite flakes).
RGB sequence of colors: red matches the first element in a row, green – second, blue - third.

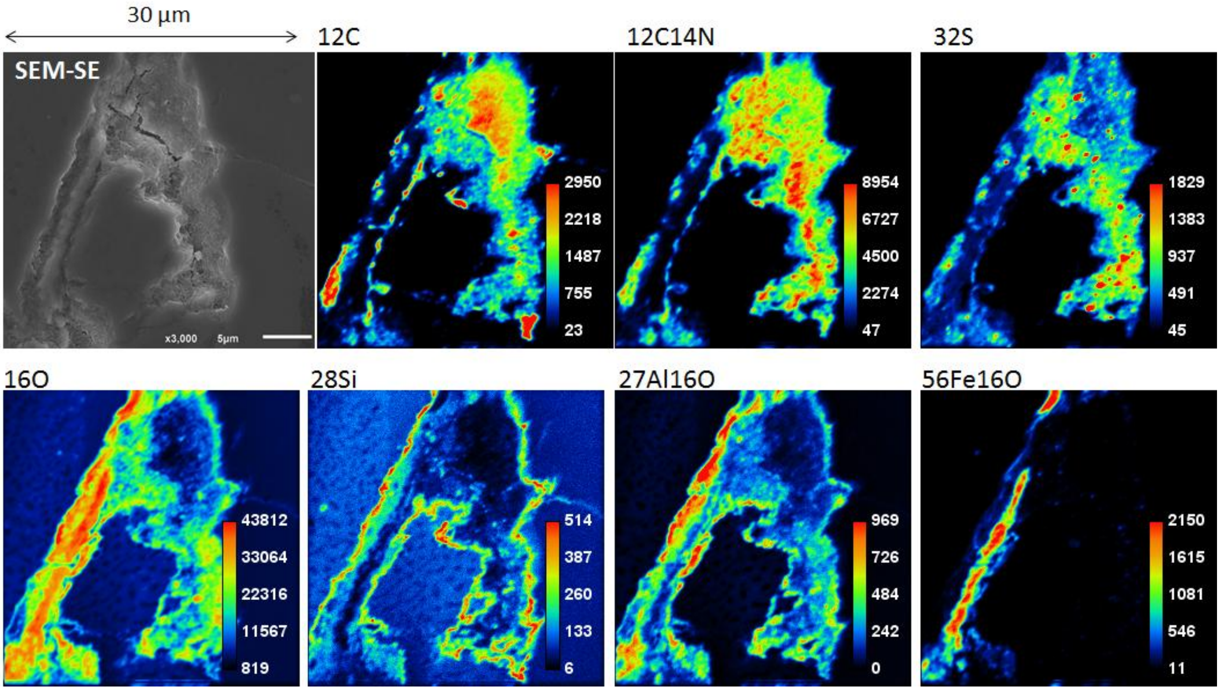

Figure S4. Visualization of organic matter attributed to the etching pit, Fe-coated hyphae crosses the pit (NanoSIMS data)

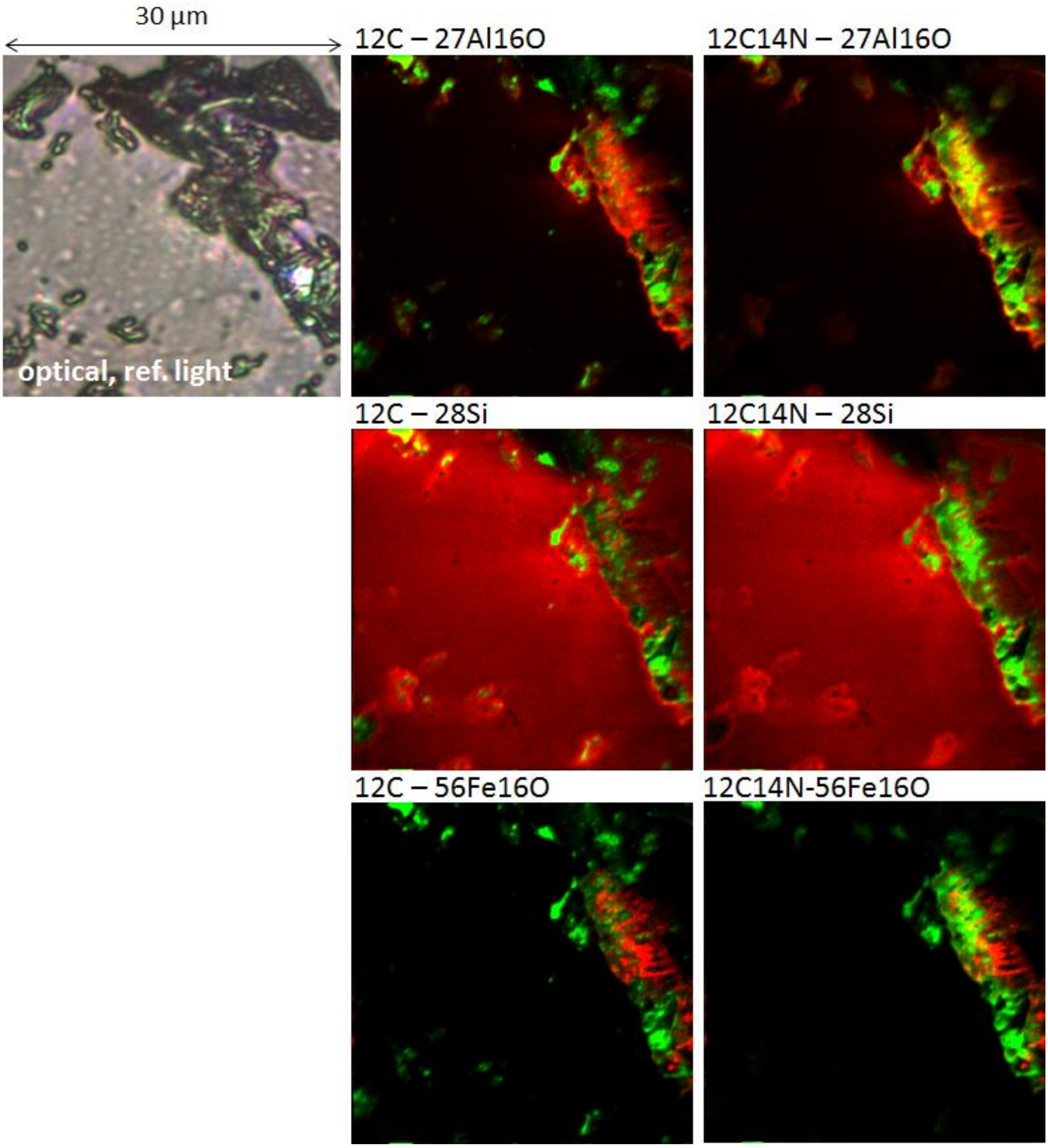

Figure S5. Visualization of organic matter attributed to the etching pits. Composite image, the first element in pair is imaged in red, the second – in green (NanoSIMS data).

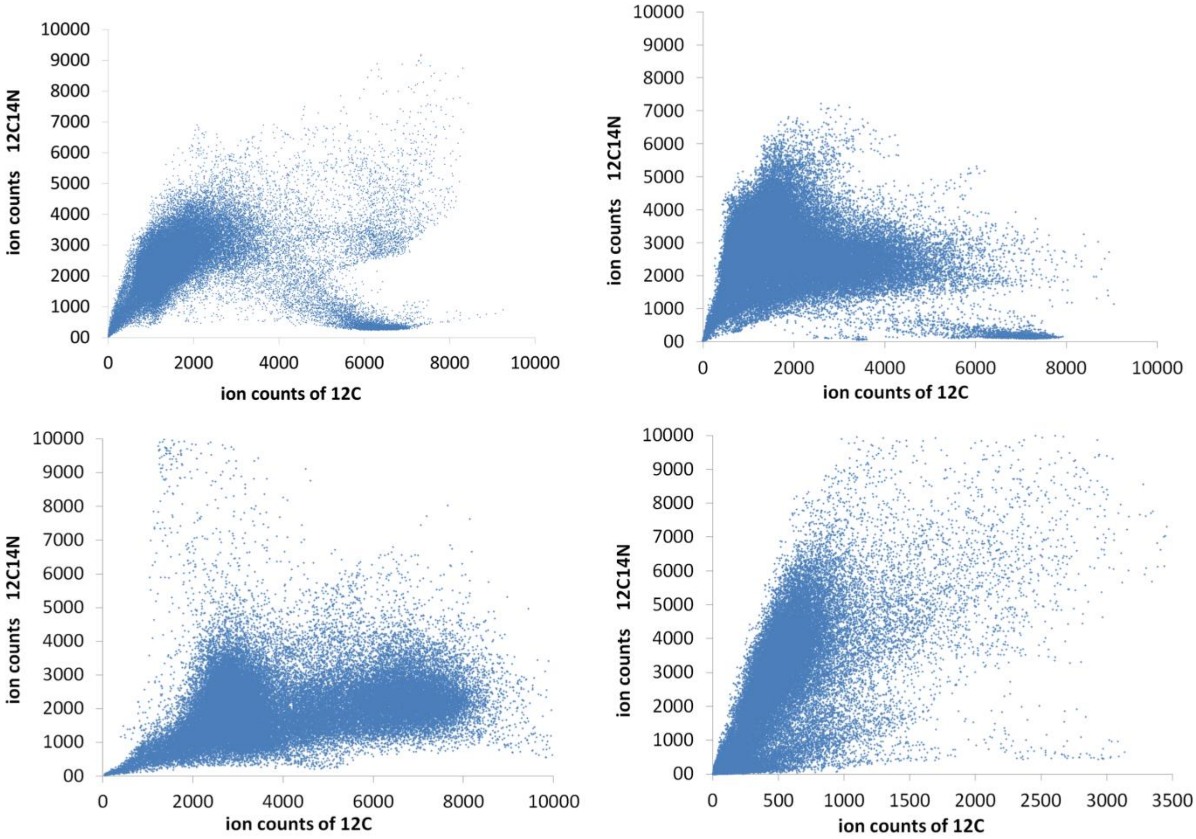

Figure S6. Scatter plots of the secondary ions counts ($^{12}C^{-}$ and $^{12}C^{14}N^{-}$) reveal several different organic matter generations that are present in parallel in endolithic organo-mineral horizons (NanoSIMS data).

Secondary ions colocalization in endolithic organo-mineral horizon from the Larsemann Hills, East Antarctica

(NanoSIMS data, correlation matrix upon *Pearson's r* between ions counts)

	$^{12}C^-$	$^{16}O^-$	$^{12}C^{14}N^-$	$^{28}Si^-$	$^{32}S^-$	$^{27}Al^{16}O^-$	$^{56}Fe^{16}O^-$
$^{12}C^-$	1,00	0,01	0,85	0,33	0,62	0,16	-0,12
$^{16}O^-$	0,01	1,00	0,31	0,47	0,52	0,90	0,66
$^{12}C^{14}N^-$	0,85	0,31	1,00	0,46	0,86	0,52	0,10
$^{28}Si^-$	0,33	0,47	0,46	1,00	0,30	0,61	-0,11
$^{32}S^-$	0,62	0,52	0,86	0,30	1,00	0,65	0,49
$^{27}Al^{16}O^-$	0,16	0,90	0,52	0,61	0,65	1,00	0,54
$^{56}Fe^{16}O^-$	-0,12	0,66	0,10	-0,11	0,49	0,54	1,00

Chemical composition of organic matter in endolithic system from the Larsemann Hills, East Antarctica according to the 13C-CPMAS NMR

Organic matter generations	Chemical shift regions under consideration of spinning side bands, proportion [%]				
	Alkyl	O/N-Alkyl	Aryl	Carboxyl	Alkyl / O/N-Alkyl
Brown varieties	8,69	80,66	2,66	7,92	0,11
Green varieties	18,30	56,48	11,69	12,41	0,32
